# Unexpected vascular structure during rectus sheath block in a cirrhotic patient

**DOI:** 10.1186/s40981-026-00848-6

**Published:** 2026-02-06

**Authors:** Tatsumi Yakushiji, Jun Honda, Keisuke Yoshida, Satoki Inoue

**Affiliations:** Department of Anesthesiology, Fukushima Medical University, Fukushima, Japan

To the editor,

We present a case of liver cirrhosis with extensive collateral circulation that complicated regional anesthesia, similar to the case described by Yoshida et al. [1].

The patient was a woman in her 70 s who was referred for resection of a giant ovarian tumor. She had hepatocellular carcinoma arising from liver cirrhosis, which was classified as Child–Pugh class B with thrombocytopenia (platelets 30,000/µL). Due to the tumor’s　size,　open abdominal surgery was planned under general and regional anesthesia. Given the thrombocytopenia, a rectus sheath block was chosen instead of epidural anesthesia.

However, during ultrasound-guided rectus sheath block, dilated vascular structures were noted within the left rectus abdominis muscle (Fig. 1 A). Color Doppler ultrasound confirmed vascular flow, suggesting that these were dilated veins ༈Fig. 1B༉. We therefore aborted the rectus sheath block and performed a lateral transversus abdominis plane block instead. A subsequent review of the preoperative CT revealed dilation of the abdominal wall veins running through the rectus abdominis muscle　(Fig. 2).

**Fig. 1:**
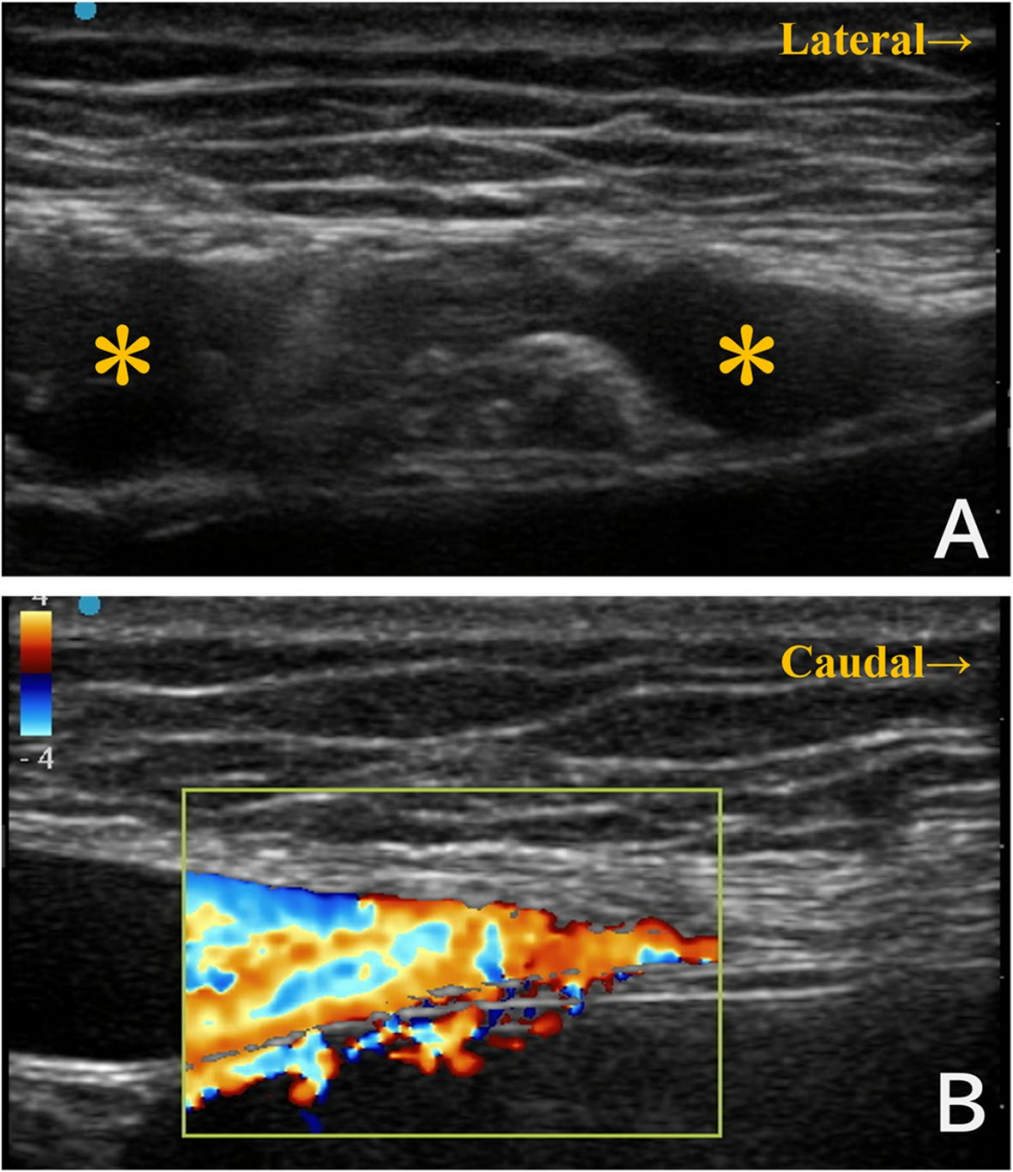
Ultrasound findings during attempted rectus sheath block. (**A**) Axial ultrasound image showing vascular structures (＊) within the left rectus abdominis muscle. (**B**) Sagittal Doppler ultrasound confirmed vascular flow

**Fig. 2:**
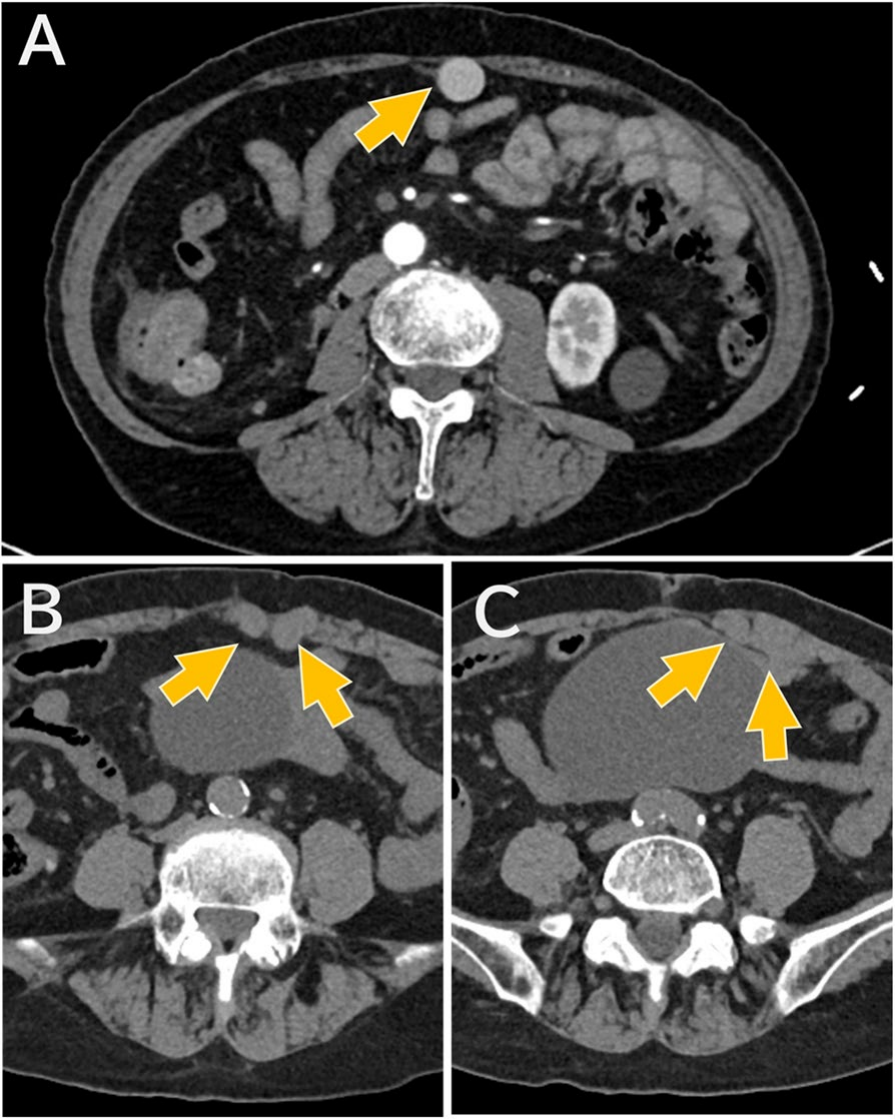
Preoperative CT images showing dilated abdominal wall veins. (**A**) Enhanced CT image showing a prominent collateral vein (arrows) near the abdominal wall. (**B**, **C**) Unenhanced CT images at progressively caudal levels, demonstrating the course of the dilated vein (arrows) as it runs through the rectus abdominis muscle.※Contrast-enhanced images are not available for these slices

This case highlights two clinical considerations. First, in patients with liver cirrhosis, unexpected development of collateral vessels may pose risks during regional anesthesia. Vascular spiders are a well-known venous anomaly seen in liver cirrhosis. They are usually prominent on the surface of the abdominal wall, allowing us to avoid the risk of vascular puncture by visual inspection. However, as in this case, collateral vessels may exist in unexpected locations and may not be visible on the surface. If preoperative imaging is available, careful evaluation for abnormal collateral pathways is recommended. Such vascular risks are not limited to cirrhosis. Araz et al. reported a case of abdominal wall hematoma following a TAP block, caused by injury to a branch of the inferior epigastric artery [2]. Second, clinicians should be prepared with alternative analgesic strategies in case the initially planned block cannot be performed. Although local infiltration anesthesia was a theoretical option in this case, we considered the likelihood of aberrant vessels around the surgical site and chose to modify our approach accordingly. These vessels can be markedly dilated and therefore vulnerable not only to needle injury but also to surgical trauma. In the presence of cirrhosis-related coagulopathy, problematic hemorrhage may occur. The planned surgical approach, as well as any analgesic procedures, should be re-evaluated in consultation with the surgical team.

## Data Availability

Not applicable.
